# Thermal Stability and Irradiation Resistance of (CrFeTiTa)_70_W_30_ and VFeTiTaW High Entropy Alloys

**DOI:** 10.3390/ma18051030

**Published:** 2025-02-26

**Authors:** André Pereira, Ricardo Martins, Bernardo Monteiro, José B. Correia, Andrei Galatanu, Norberto Catarino, Petra J. Belec, Marta Dias

**Affiliations:** 1 Instituto de Plasmas e Fusão Nuclear (IPFN), Instituto Superior Técnico, Universidade de Lisboa, Campus Tecnológico e Nuclear, Estrada Nacional 10, 2695-066 Bobadela, Portugal; andre.sampaio.pereira@tecnico.ulisboa.pt (A.P.); ricardo.martins@ctn.tecnico.ulisboa.pt (R.M.); bernardo.monteiro@ctn.tecnico.ulisboa.pt (B.M.); 2 Laboratório Nacional de Energia e Geologia (LNEG), Estrada do Paço do Lumiar, 1649-038 Lisboa, Portugal; brito.correia@lneg.pt; 3 National Institute of Materials Physics, Atomistilor Street 405 A, 077125 Magurele, Ilfov, Romania; gala@infim.ro; 4 Instituto de Plasmas e Fusão Nuclear (IPFN), Departamento de Engenharia e Ciências Nucleares (DECN), Instituto Superior Técnico, Universidade de Lisboa, Campus Tecnológico e Nuclear, Estrada Nacional 10, 2695-066 Bobadela, Portugal; norberto.catarino@ctn.tecnico.ulisboa.pt; 5 Department for Nanostructured Materials, Jožef Stefan Institute, Jamova cesta 39, 1000 Ljubljana, Slovenia; petra.jenus@ijs.si

**Keywords:** high-entropy alloy, nuclear fusion, microstructure, thermal barrier, irradiation

## Abstract

Nuclear fusion is a promising energy source. The International Thermonuclear Experimental Reactor aims to study the feasibility of tokamak-type reactors and test technologies and materials for commercial use. One major challenge is developing materials for the reactor’s divertor, which supports high thermal flux. Tungsten was chosen as the plasma-facing material, while a CuCrZr alloy will be used in the cooling pipes. However, the gradient between the working temperatures of these materials requires the use of a thermal barrier interlayer between them. To this end, refractory high-entropy (CrFeTiTa)_70_W_30_ and VFeTiTaW alloys were prepared by mechanical alloying and sintering, and their thermal and irradiation resistance was evaluated. Both alloys showed phase growth after annealing at 1100 °C for 8 days, being more pronounced for higher temperatures (1300 °C and 1500 °C). The VFeTiTaW alloy presented greater phase growth, suggesting lower microstructural stability, however, no new phases were formed. Both (as-sintered) alloys were irradiated with Ar^+^ (150 keV) with a fluence of 2.4 × 10^20^ at/m^2^, as well as He^+^ (10 keV) and D^+^ (5 keV) both with a fluence of 5 × 10^21^ at/m^2^. The morphology of the surface of both samples was analyzed before and after irradiation showing no severe morphologic changes, indicating high irradiation resistance. Additionally, the VFeTiTaW alloy presented a lower deuterium retention (8.58%) when compared to (CrFeTiTa)_70_W_30_ alloy (14.41%).

## 1. Introduction

The reliance of society on fossil fuels for electricity generation has led to an increase in greenhouse gas emissions. With the expected population growth in the coming decades and the expected increase in electrical energy consumption, particularly in developing countries, it is necessary to find clean and reliable energy sources. Nuclear fusion reactors, in which the fusion of light atoms generates energy [1], are a promising technology. The fusion of deuterium (D) and tritium (T) is currently considered the best option for future commercial thermonuclear fusion reactors [2]. In this reaction, α particles and a neutron with 3.5 MeV and 14.1 MeV, respectively, are produced. Over the last decades, there has been an increased international effort in research and development in this field; one of the most promising projects is the International Thermonuclear Experimental Reactor (ITER) [3]. This project aims to build the largest tokamak ever to achieve fusion conditions for extended periods to prove the viability and safety of nuclear fusion, test technologies, and materials, and define the design of a future demonstration power plant (DEMO) [4,5].

In this context, one of the biggest challenges is the development of structural and functional materials that can withstand the conditions inside the reactor: high temperature, high heat flux, and intense flux of high energy neutrons [5]. The diverter, located at the bottom of the plasma chamber, where the thermal power from the plasma is exhausted [4], is one of the most challenging areas. Tungsten (W), with a high melting temperature, low sputtering rates, low neutron activation, and low tritium and deuterium retention [6,7], was chosen as the plasma-facing material. CuCrZr alloy was chosen as the heat sink due to its high thermal conductivity and strength. However, there is a gap between the operating temperatures of these two materials. Tungsten presents a high ductile–brittle temperature transition (DBTT) [8], which requires a high-temperature operation, while CuCrZr alloy presents relatively low working temperatures (180–350 °C) [6], with loss of ductility caused by irradiation embrittlement at lower temperatures and loss of mechanical strength at higher temperatures due to over-aging [9,10]. Therefore, it is necessary to use a thermal barrier interlayer in-between these materials, allowing each of them to operate at their respective working temperatures.

Previous studies on (CrFeTiTa)_70_W_30_ and VFeTiTaW alloys have already demonstrated notable mechanical properties as candidates for the thermal barrier interlayer [11]; for example, showing a ductile behavior strain–-stress magnitude of around 300 MPa for vanadium and around 400 MPa for chromium-based alloys, exhibited at 1000 °C. Nevertheless, there is still a lack of knowledge on thermal stability at higher temperatures and irradiation resistance, which are important aspects to consider for the behavior of the materials subject to extreme environments, especially nuclear fusion. Refractory, low activation high-entropy alloys such as TiVCrTa [10], TiVCrFeMn [12], and (CrVTiTa)_1−x_W_x_ [13], where x is the atomic fraction (0.3 to 0.9), have been proposed for nuclear applications. This class of alloys is particularly promising as a thermal barrier interlayer due to its high thermal stability [14] and high temperature strength [15,16], as well as high radiation resistance [17] and low thermal diffusivity [18]. Based on this, the study of refractory alloys was pursued based on W, since it is the chosen plasma-facing component. This work presents the production of entropy (CrFeTiTa)_70_W_30_ and VFeTiTaW alloys by mechanical alloying and sintering together with their thermal and irradiation resistance. Scanning electron microscopy (SEM) coupled with energy-dispersive X-ray spectroscopy (EDS), as well as X-ray diffraction, will be used to define the structure of the materials before and after the irradiation or annealing. Moreover, Nuclear Reaction Analysis (NRA) will be used to measure the deuterium retention.

## 2. Materials and Methods

Fe, Ti, Ta, Cr, V, and W powders with a nominal purity of at least 99.5% and with an average particle size of 10 μm (AlfaAesar) were mixed in mill jars in the adequate proportion to produce the (CrFeTiTa)_70_W_30_ and VFeTiTaW alloys, in a glove box under an argon atmosphere to avoid oxidation. The resulting powder mixtures were mechanically alloyed in a high-energy planetary ball mill (PM 400 MA) using tungsten carbide (WC) balls and mill jars, with a ball-to-powder ratio of 10:1, at 350 rpm for 2 h, without a process control agent. The milled powders were consolidated by spark plasma sintering (SPS) in an FCT System GmbH equipment (Freiberg, Germany) using graphite molds (12 mm diameter). The sintering process was performed at 1150 °C with an applied force of 9 kN and a holding time of 5 min under vacuum.

The sintered samples were ground with a coarse SiC paper (P180) in order to remove the residues left by the graphite mold used in the consolidation process. After that, the samples were ground with increasingly finer SiC paper (up to P4000) and polished first with diamond suspensions (3 µm and 1 µm), followed by a colloidal silica suspension (OPS) with an average particle size of 0.04 µm. To evaluate the evolution of their microstructure at high temperatures, samples of both compositions were annealed at 1100 °C, 1300 °C, and 1500 °C for 8 days under a vacuum atmosphere.

A Bruker D8 AXS diffractometer with Cu Kα1 and Cu Kα2 radiation equipped with a Göbel mirror and a Soller slit was used to perform the X-ray diffraction of the consolidated samples before and after annealing and irradiation. Due to the reduced depth of the implanted region, the irradiated samples were analyzed using near-grazing angle incidence X-ray diffraction (GIXRD) with an incident angle of 3°. Since the intention is to compare these samples with the nonirradiated ones, these samples were also analyzed with this geometry. The phases were identified by comparing the experimental diffractogram with simulated XRD patterns, using the PowderCell software version 2.0 [19]. The Pearson’s crystal database [20] was used to obtain crystallographic information for phase comparation.

The microstructures of annealed samples were studied by scanning electron microscopy (SEM) in backscattering electron (BSE) mode, using a Thermo Scientific^TM^ Phenon^TM^ ProX G6 scanning electron microscope (Waltham, MA, USA) with a 15 keV electron beam. To analyze the morphologic changes after irradiation, secondary electron images (SE) of the as-sintered and irradiated samples were obtained with the same equipment. The SE images were taken in a flat and tilted (70°) configuration. EDS was used for elemental mapping and quantitative determination of phase composition. The quantitative analysis was performed on samples of both alloy systems annealed at 1100 °C, 1300 °C and 1500 °C, and each of the phases was analyzed in at least 10 randomly chosen points, in which the phase to be analyzed is large enough so that there is no contribution from neighboring phases.

In order to reproduce the D-T fusion reaction effects on the produced alloys, the materials were subjected to room temperature implantation of He^+^ (10 keV) followed by implantation of D^+^ (5 keV) to a fluence of 5 × 10^21^ at/m^2^ for both ions. Moreover, the effects of 14 MeV energetic neutrons, which result from the D-T fusion reaction, will be experimentally simulated by irradiation of 150 keV Ar^+^ ions to a fluence of 2.4 × 10^20^ at/m^2^ at room temperature. This surrogate for neutron damage is currently a standard option, validated by the comparison of the radiation-induced defect structures in steels by heavy ions vs. neutrons [21,22]. The fluence of the Ar^+^ implantation was chosen to cause displacement damage of ~100 dpa, which corresponds to the neutron damage caused during 5 years of DEMO operation [23]. Table 1 shows the implantations performed in each sample. 

The implantation energies were chosen so that the He^+^ and D^+^ implantation profiles overlap with the damage profile caused by Ar^+^ implantation. The fluence and energies were determined through simulations performed using SRIM [24]. Regarding the Ar^+^ fluence, the simulation results showed that for an energy of 150 keV, a fluence of 2.3 × 10^20^ at/m^2^ and 2.5 × 10^20^ at/m^2^ is required to obtain a maximum damage of 100 dpa in the (CrFeTiTa)_70_W_30_ sample and in the VFeTiTaW sample, respectively. Moreover, the zone where D+ and He+ are implanted overlaps with the zone damaged by Ar^+^ implantation.

Since both alloys were irradiated with Ar^+^ simultaneously, an intermediate value was chosen (2.4 × 10^20^ at/m^2^). The affected zone is limited to around 150 nm. The irradiations were performed sequentially (1-Ar^+^, 2-He^+^, 3-D^+^) in a Danfysik 1900 implanter (Taastrup, Denmark). To investigate deuterium retention on the irradiated samples, the NRA technique using the ^2^H(^3^He,p_0_)^4^He reaction was used to measure deuterium depth profiles. A 1750 keV ^3^He^+^ beam was used. The analysis of the obtained spectra was performed using SIMNRA software (https://mam.home.ipp.mpg.de/) [25], and the cross-section data used in this analysis was retrieved from the IBANDL database [26].

## 3. Results and Discussion

### 3.1. Structural Characterization

**Figure 1:**
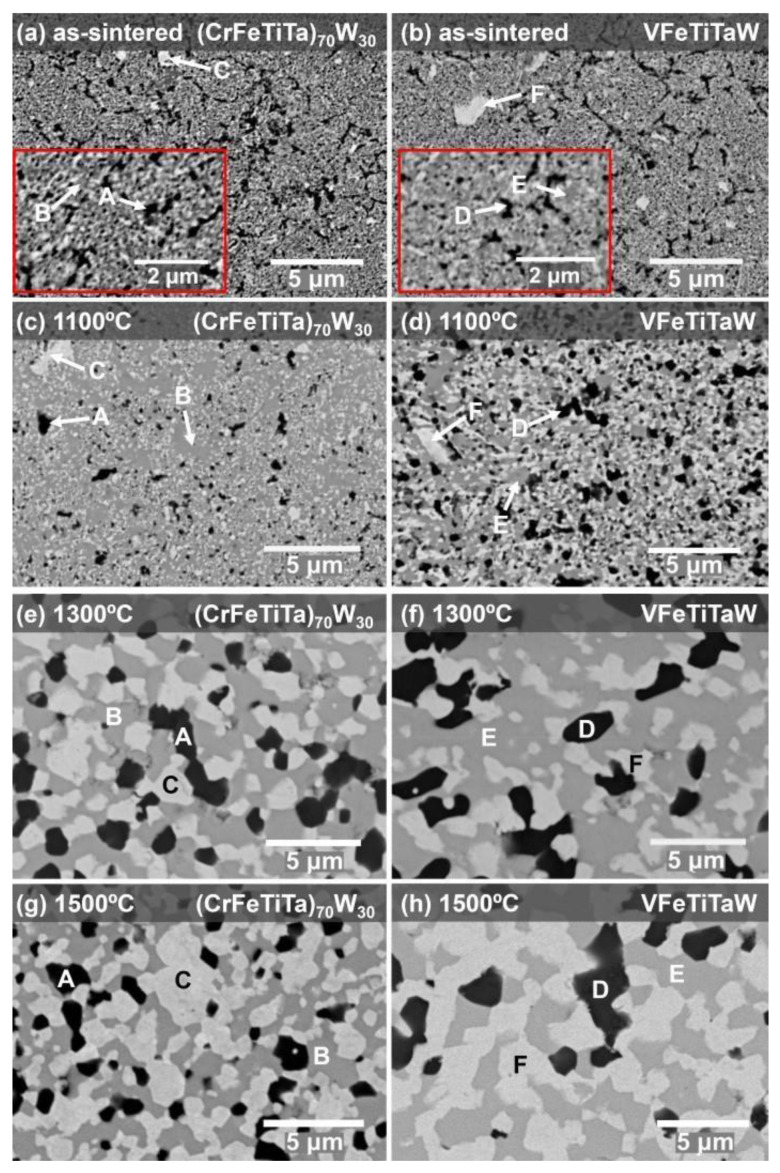
SEM images collected in BSE mode showing the microstructure of (CrFeTiTa)_70_W_30_ alloy as-sintered (**a**) and annealed at 1100 °C (**c**), 1300 °C (**e**) and 1500 °C (**g**); VFeTiTaW alloy as-sintered (**b**) and annealed at 1100 °C (**d**), 1300 °C (**f**) and 1500 °C (**h**).

Figure 1a and Figure 1b show the microstructure of the (CrFeTiTa)_70_W_30_ alloy and the VFeTiTaW alloy after sintering, respectively. Both alloys present a fine microstructure (submicrometric), composed of three phases: a dark phase (**A**; **D**), an intermediate phase (**B**; **E**), and a lighter phase (**C**; **F**), homogeneously distributed. Figure 1c–h shows the microstructures of both alloy systems annealed at 1100 °C, 1300 °C, and 1500 °C for 8 days. All annealed samples present the same number of phases as the as-sintered samples, indicating that no reactions in the solid state occurred. Both (CrFeTiTa)_70_W_30_ and VFeTiTaW alloys showed phase growth after annealing at 1100 °C up to 1500 °C for 8 days. However, the system containing vanadium shows faster growth, suggesting less stability of its microstructure. As the annealing temperature increases, more pronounced phase growth is observed which has occurred due to the globalization of the phases, promoted by an increase in the diffusion of the elements at higher temperatures.

**Figure 2:**
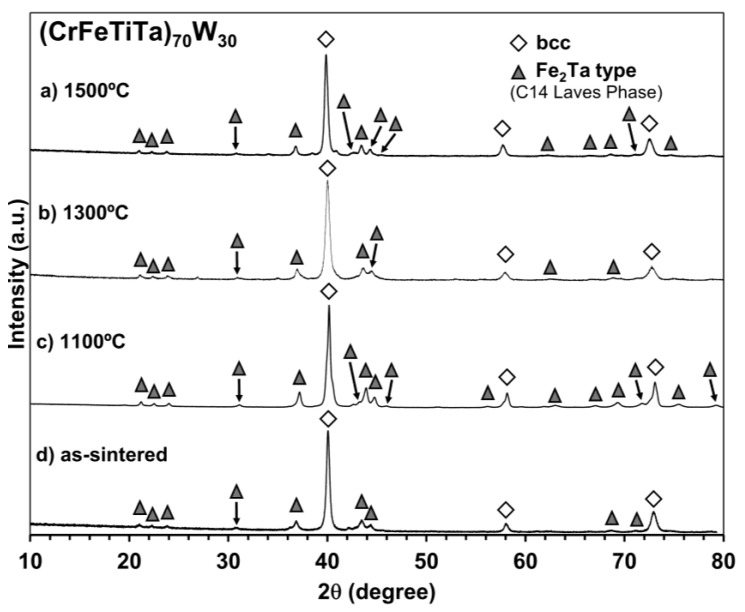
Diffractogram of (CrFeTiTa)_70_W_30_ alloy annealed at 1500 °C (**a**), 1300 °C (**b**) and 1100 °C (**c**), and as-sintered (**d**). The symbol shows the presence of a bcc-type structure and the symbol represents the Fe_2_Ta phase.

Figure 2 shows the diffractograms of the (CrFeTiTa)_70_W_30_ alloy annealed at 1500 °C (a), 1300 °C (b), and 1100 °C (c), as well as the diffractogram of the as-sintered sample (d). After sintering, peaks with higher intensity corresponding to a bcc-type structure are observed with a lattice parameter of a = 3.17 nm. In comparison to the bcc-type structure peaks identified in the milled powder, presented in reference [11], the as-sintered samples display sharper peaks and shifted to lower values of 2θ (milled presented a = 3.15 nm and as-sintered a = 3.17 nm), which is an indication of an increase in crystallinity and lattice expansion. These changes are in accordance with the diffusion of the elements at higher temperatures inferred above in the microstructure studies during the sintering process. Furthermore, after sintering, the minor peak corresponding to unmixed Ta in the milled powder [11] is no longer visible, indicating that the elevated temperatures during sintering allowed the diffusion of Ta and its incorporation into other phases. Since the atomic radius of Ta is larger than that of the other elements, its incorporation into the bcc-type structure can lead to an increase in the lattice parameter, which can explain the observed shift. The peaks corresponding to tungsten carbide (WC) are also no longer visible. However, it is important to note that the carbon previously present in the WC in the milled powder remains, and it might be integrated into one or more phases as a contaminant. Additionally, a new phase was formed after sintering since it is possible to identify new peaks which correspond to a phase with the Fe_2_Ta-type structure, the C14 hexagonal Laves phase. No considerable changes in the diffractograms were observed after annealing at all the temperatures, which suggests high thermal phase stability.

**Figure 3:**
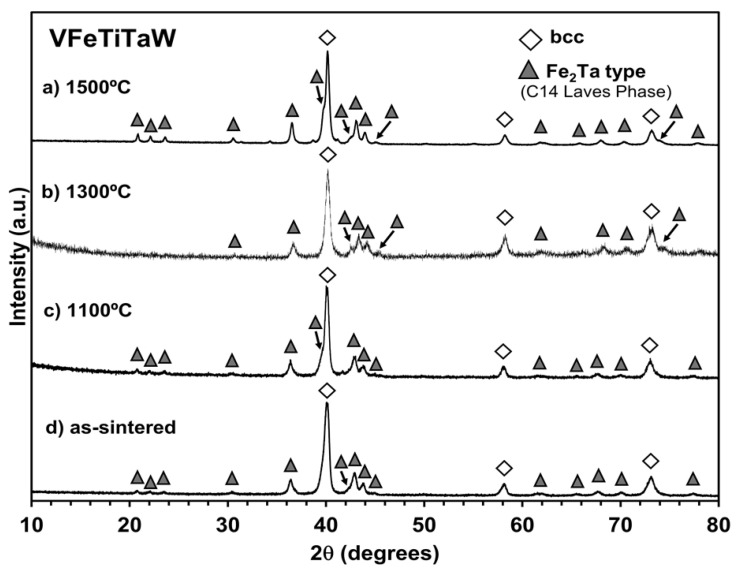
Diffractogram of VFeTiTaW alloy annealed at 1500 °C (**a**), 1300 °C (**b**) and 1100 °C (**c**), and as-sintered (**d**). The symbol shows the presence of a bcc-type structure, and the symbol represents the Fe_2_Ta phase.

Like the (CrFeTiTa)_70_W_30_ alloy, the VFeTiTaW alloy presents, after sintering, peaks corresponding to the bcc type structure and peaks corresponding to a new C14 Laves phase ascribed to Fe_2_Ta, as shown in Figure 3d. Additionally, in this case, no considerable changes were observed in the diffractograms after annealing, indicating high thermal stability. Thus, during annealing, there was no phase transformation, only their growth. Several authors have reported the formation of Laves phases (C14 or C15) in refractory high entropy alloys [11,27], usually associated with the presence of elements such as Cr or V [27,28]. Laves phases are intermetallic compounds with AB_2_ (or B_2_A) stoichiometry, where the atoms with bigger atomic radius (such as W, Ti, Mo, Zr, Ta) occupy the A sites while the smaller atoms (such as Cr, Fe, V) occupy the B sites [29]. The formation of these Laves phases is promoted by large negative mixing enthalpies and significant atomic size mismatch between the elements [30,31].

**Figure 4:**
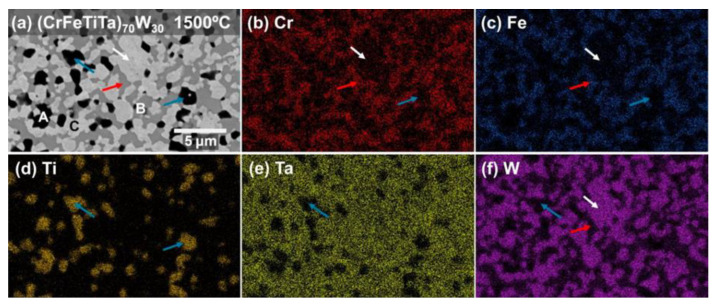
(**a**) SEM image collected in BSE mode of (CrFeTiTa)_70_W_30_ alloy annealed at 1500 °C for 8 days, and EDS maps for (**b**) Cr-Lα, (**c**) Fe-Lα, (**d**) Ti-Kα, (**e**) Ta-Lα and (**f**) W-Lα.

**Figure 5:**
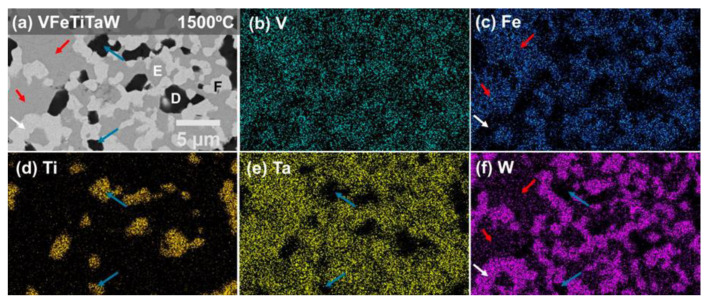
(**a**) SEM image collected in BSE mode of VFeTiTaW alloy annealed at 1500 °C for 8 days, and EDS maps for (**b**) V-Lα, (**c**) Fe-Lα, (**d**) Ti-Kα, (**e**) Ta-Lα and (**f**) W-Lα.

In order to discover how the elements are distributed and to determine the composition of each phase, EDS analysis was performed. Figure 4 and Figure 5 present the EDS maps obtained on the (CrFeTiTa)_70_W_30_ sample annealed at 1500 °C, and on the VFeTiTaW sample annealed at 1500 °C, respectively.

In the (CrFeTiTa)_70_W_30_ sample, Figure 4, the darker phase (phase **A**, indicated with blue arrows in Figure 4a) is titanium-rich and depleted in the remaining elements. The EDS point analysis revealed the presence of oxygen in this phase, indicating that it can be Ti-rich oxide. The intermediate phase, or phase **B** (indicated by the red arrow in Figure 4a), is a Cr-Fe-Ta-rich phase but is depleted in W (only 7.7 at.%) and seems to have no Ti. Finally, the lighter phase (phase **C**) (indicated by the white arrow in Figure 4a) is W-rich (76.3 at.%) together with Ta (21.1 at.%). The phase with the bcc-type structure identified in the diffractogram of the (CrFeTiTa)_70_W_30_ alloy (Figure 2) has a lattice parameter very similar to that of W (which also has a bcc-type structure). So, it probably corresponds to the phase rich in this element (phase **C**). Thus, phase **C** is possibly a solid solution of W and Ta with a bcc-type structure.

Another phase identified in the diffractogram (Figure 2) was a C14-type Laves phase. Phase **A** has already been identified as a titanium-rich oxide, and phase **C** as a bcc-type structure with W and Ta elements. Thus, the Laves phase identified must correspond to phase **B**. The phase used to simulate the structure of this phase and compare it with the peaks of the experimental diffractograms was Fe_2_Ta. The experimental peaks are slightly shifted to smaller values of 2θ with respect to the Fe_2_Ta, which means that the Laves phase present in the sample has larger lattice parameters than that of the simulated Fe_2_Ta phase. This suggests that its composition does not exactly correspond to that of Fe_2_Ta (66.6 at.% Fe + 33.3 at.% Ta), but larger atoms have been dissolved. The composition of phase **B** of the (CrFeTiTa)_70_W_30_ sample annealed at 1500 °C, 49 at.% Fe, 20.8 at.% Ta; 22.5 at.% Cr and 7.6 at.% W suggests that it is a phase derived from Fe_2_Ta, in which part of the Fe and Ta atoms were replaced by Cr and W atoms. So, phase **B** must be a B_2_A multi-element (Cr,Fe)_2_(Ta,W) C14 Laves phase.

**Figure 6:**
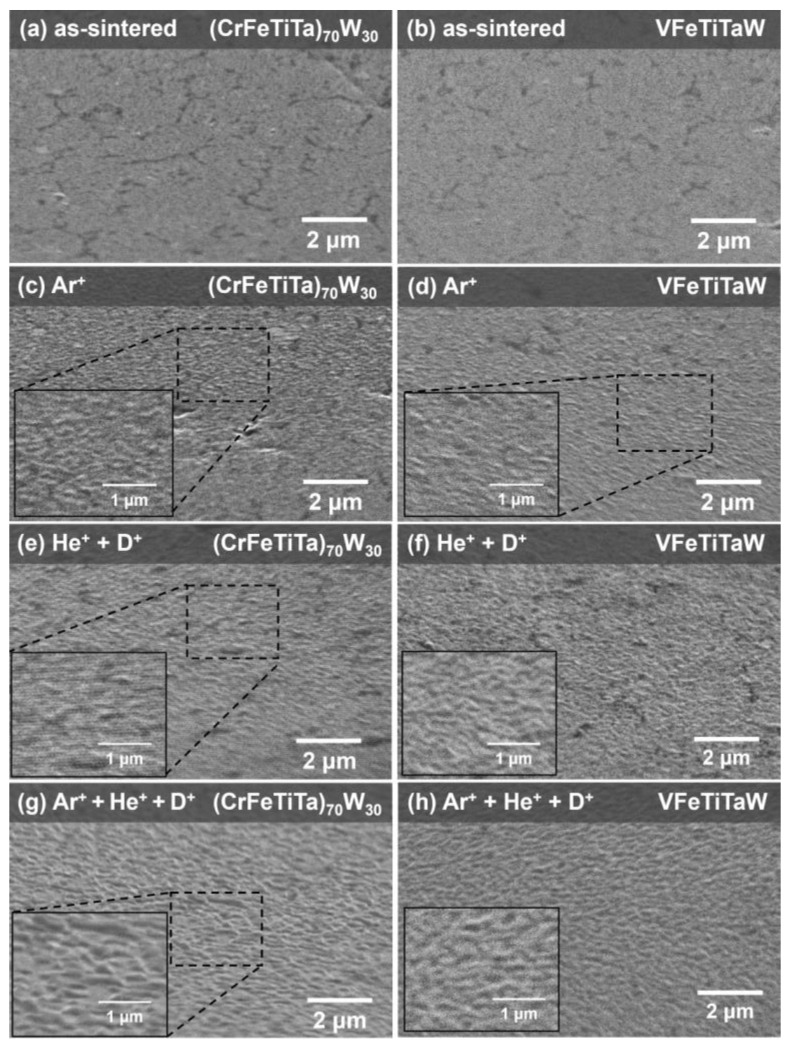
SE images taken at a tilted configuration (70°) showing the topography of the (CrFeTiTa)_70_W_30_ alloy before irradiation (**a**) and after irradiation with Ar^+^ (**c**), He^+^ + D^+^ (**e**) and Ar^+^ + He^+^ + D^+^ (**g**), and SE images taken at a tilted configuration (70°) showing the topography of the VFeTiTaW alloy before irradiation (**b**) and after irradiation with Ar^+^ (**d**), He^+^ + D^+^ (**f**) and Ar^+^ + He^+^ + D^+^ (**h**).

Similarly to phase **A**, phase **D**, the darker phase of the VFeTiTaW alloy (indicated by the blue arrows in Figure 5a), is also rich in Ti and depleted in the remaining elements. The point EDS analysis of this phase also indicated that it is rich in oxygen, which could mean that it is an oxide. Phase **E** (intermediate phase indicated by the red arrows in Figure 5a) is rich in V, Fe, and Ta, is depleted in W (only 6.7 at.%), and does not contain Ti. Phase **F** (lighter phase indicated by the white arrows in Figure 6a) is rich in W (65.1 at.%), Ta (17.2 at.%), and V (17.8 at.%). The existence of a bcc-type structure and a C14 Laves phase was also identified in the VFeTiTaW alloy. The bcc-type structure should correspond to phase **F**, whose composition (65.1 at.% W; 17.2 at.% Ta and 17.8 at.% V, determined by EDS in the VFeTiTaW sample annealed at 1500 °C) indicates that it is a solid solution of W, Ta and V. Phase **D** corresponds to an oxide rich in Ti, so the Laves phase should correspond to phase **E**.

As in the other composition, the prototype phase used to simulate the structure of this phase and compare it with the peaks of the experimental diffractograms was Fe_2_Ta. Considering its composition (40.6 at.% Fe; 25.4 at.% Ta; 27.3 at.% V and 6.7 at.% W), it probably corresponds to a phase derived from Fe_2_Ta, in which part of the atoms were replaced by V and W. As previously discussed, V occupies Fe sites (B sites), and W occupies Ta sites (A sites). Thus, phase **E** corresponds to the multi-element intermetallic (V,Fe)_2_(Ta,W) with C14 Laves phase structure. Furthermore, the ratio between the atoms in the B position (Fe and V) and the atoms in the A position (Ta and W) is 68:32, which is very close to the ideal stoichiometry of the Laves phases (66.7:33.3). Additionally, the ratio between the radii of Ta (0.143 nm) and Fe (0.124 nm) is high (1.15) and the enthalpy of mixing of these two elements (ΔHmix) is large and negative (−15 kJ/mol [32]). These two factors are the driving force that leads to the formation of the Laves phase. Furthermore, thermodynamic calculations [32] predicted the formation of a single solid solution. Although these predictions are observed for powder high entropy alloys, they might not be observed upon consolidation, as seen in [11]. The existence of multiple phases in both alloys indicates that the entropic effect was not sufficient to promote the formation of a single-phase consolidated alloy with only a disordered solid solution, and other parameters must be considered in phase prediction.

Table 2 shows the average atomic composition of phase **B** and phase **C** of the (CrFeTiTa)_70_W_30_ alloy, as well as the average composition of phases **E** and phase **F** of the VFeTiTaW alloy, determined by point EDS analysis on samples annealed at 1500 °C for 8 days.

### 3.2. Irradiation Analysis

The irradiation resistance of both as-sintered alloys was studied by irradiating the materials with Ar^+^ (150 keV) with a fluence of 2.4 × 10^20^ at/m^2^, and He^+^ (10 keV) and D^+^ (5 keV) both with a fluence of 5 × 10^21^ at/m^2^. The surface of the samples was analyzed before and after irradiation.

Figure 6 shows the topography of the (CrFeTiTa)_70_W_30_ alloy tilted 70 degrees, before implantation (a) and after implantation with Ar^+^ (c); He^+^ + D^+^ (e) and Ar^+^ + He^+^ + D^+^ (g), as well as the topography of the VFeTiTaW alloy in the same conditions.

Before implantation (Figure 6a,b), both samples present a pristine surface, with no roughness visible at this scale. After Ar^+^ implantation (Figure 6c,d), both alloys show swelling, and no fracture is observed. In the case of the samples irradiated sequentially with He^+^ followed by deuterium (D^+^), a more pronounced swelling is observed (Figure 6e,f)). Additionally, when Ar^+^ is implanted before He^+^ and D^+^, there is an even more pronounced swelling effect (Figure 6g,h)), which is a consequence of the defects caused by the Ar^+^ implantation. The swelling appears to be distributed uniformly over the sample surface; however, since the microstructure of these samples is very fine (submicrometric) it is not possible to conclude about the irradiation effect on each of the presented phases.

**Figure 7:**
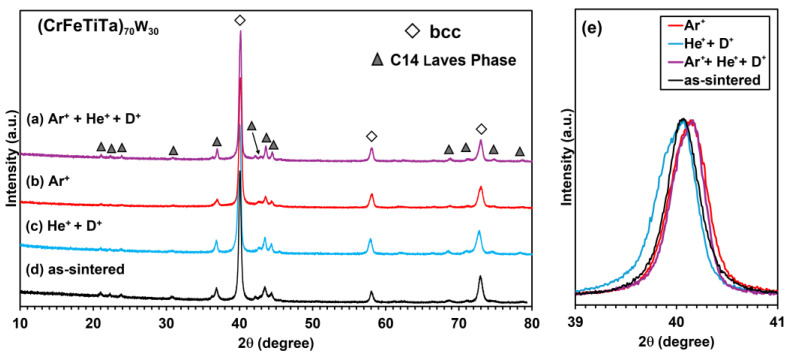
Diffractograms of (CrFeTiTa)_70_W_30_ alloy before (**d**) and after irradiation with Ar^+^ + He^+^ + D^+^ (**a**), Ar^+^ (**b**), He^+^ + D^+^ (**c**), and (**e**) enlarged view of the bcc type peak at 2θ = ~40°.

**Figure 8:**
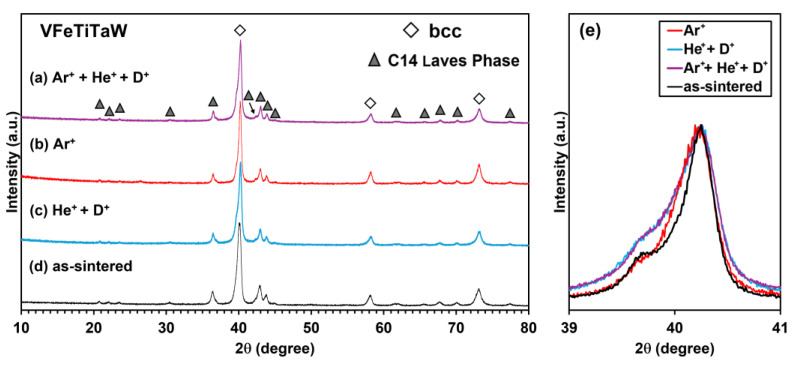
(**a**) Diffractograms of VFeTiTaW alloy before (**d**) and after irradiation with Ar^+^ + He^+^ + D^+^ (**a**), Ar^+^ (**b**), He^+^ + D^+^ (**c**), and (**e**) enlarged view of the bcc type peak at 2θ~40°.

Figure 7 and Figure 8 show the diffractograms of the (CrFeTiTa)_70_W_30_ and VFeTiTaW alloys implanted with Ar^+^ + He^+^ + D^+^ (a), Ar^+^ (b), and He^+^ + D^+^ (c), as well as the diffractogram before irradiation (d).

In both cases, a bcc-type structure and another phase with a C14 Laves phase structure continued to be identified. This indicates high stability and resistance to irradiation, at least under the tested conditions.

Figure 7e and Figure 8e show an enlarged view of the main peak of the bcc-type structure of both samples (2θ = ~40°) before [11] and after irradiation. The peaks are normalized and superimposed in order to highlight the changes caused by irradiation. In the case of the (CrFeTiTa)_70_W_30_ alloy (Figure 7e), a shift to the right is observed after implantation with Ar (Ar^+^ or Ar^+^ + He^+^ + D^+^). When the alloy is implanted with He^+^ + D^+^, a broadening of the peak on the left side (lower 2θ) was observed. This broadening is probably due to the occupation of lattice interstitial sites with helium (and deuterium) atoms [33], which can lead to lattice distortion and, consequently, to broadening of the peaks. Another factor that may be responsible for the broadening of the peaks is deuterium trapping in intrinsic and irradiation-induced defects (induced by Ar^+^ and He^+^ implantation) and subsequent deuterium agglomeration in clusters [34]. The shift to the right side (higher 2θ) can be explained by a possible lattice contraction due to the introduction of irradiation-induced compressive stresses after irradiation with heavy ions such as Ar^+^ [35,36].

In the case of the diffractograms of the VFeTiTaW alloy presented in Figure 8e, no peak shift was observed. Only broadening to the left side is observed after He^+^ + D^+^ implantation and Ar^+^ + He^+^ + D^+^ implantation.

**Figure 9:**
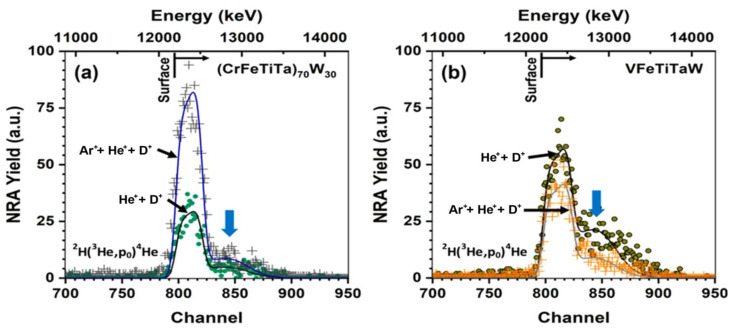
NRA curves of (CrFeTiTa)_70_W_30_ alloy (**a**) and VFeTiTaW alloy (**b**) implanted with He^+^ + D^+^ and Ar^+^ +He^+^ + D^+^.

The NRA technique was used to determine deuterium retention in samples of both alloys irradiated with He^+^ + D^+^ and Ar^+^ + He^+^ + D^+^, and the graphs are presented in Figure 9 together with the retention values, which are shown in Table 3. The (CrFeTiTa)_70_W_30_ sample irradiated with Ar^+^ + He^+^ + D^+^ presents a higher yield of the ^2^H(^3^H,p_0_)^4^He reaction compared with the sample implanted only with He^+^ + D^+^ (Figure 9a). The results indicate that there are more deuterium atoms retained when Ar is present. In fact, Ar^+^ implantation before He^+^ and D^+^ implantation led to an increase in deuterium retention from 5.47% to 14.41% (Table 3). The increase in deuterium retention with prior Ar^+^ implantation can be attributed to the creation of additional defects due to Ar^+^ irradiation, the so-called irradiation-induced defects, such as vacancies and dislocations [35]. These defects, along the intrinsic defects (such as grain boundaries), are places where deuterium (D) can be trapped. In the case of the VFeTiTaW alloy (Figure 9b), the opposite trend is observed. The VFeTiTaW sample irradiated with Ar^+^ + He^+^ + D^+^ shows lower deuterium retention (8.58%) than the sample irradiated only with He^+^ + D^+^ (13.2%). Additionally, there are tails on the right side of the NRA curves of all samples (indicated by blue arrows in Figure 9a,b), which indicate that there was diffusion of deuterium further into the sample after irradiation. Comparing the samples of (CrFeTiTa)_70_W_30_ and VFeTiTaW implanted with Ar^+^ + He^+^ + D^+^, it is observed that the VFeTiTaW alloy presents lower deuterium retention, which represents an advantage in nuclear fusion applications.

## 4. Conclusions

(CrFeTiTa)_70_W_30_ and VFeTiTaW alloys were produced by mechanical alloying and sintering and annealed at 1100 °C, 1300 °C, and 1500 °C. The irradiation resistance of the as-sintered materials was also evaluated. Therefore, the materials were irradiated with Ar^+^ (150 keV) with a fluence of 2.4 × 10^20^ at/m^2^, as well as He^+^ (10 keV) and D^+^ (5 keV), both with a fluence of 5 × 10^21^ at/m^2^. A very fine and homogeneous microstructure composed of three phases was observed in both as-sintered alloys. After annealing, considerable phase growth was observed. Both alloys presented three phases: a phase with a bcc-type structure, a hexagonal C14 Laves phase, and a Ti-rich oxide. These phases maintained their structure even after 8 days at 1500 °C, indicating high thermal stability. It was concluded that for the sample containing Cr, the bcc-type phase is a solid solution of W and Ta, while in the case of the sample containing V, it is a solid solution of W, Ta and V. In both alloys, the Laves phase is derived from Fe_2_Ta, in which Ta atoms are partially replaced by W atoms and the Fe atoms are partially replaced by Cr or V in the (CrFeTiTa)_70_W_30_ alloy and VFeTiTaW alloy, respectively. Even after high-temperature annealing (where the entropic effect is more pronounced), the existence of multiple phases persists. Both samples suffered a swelling effect after irradiation, which was more significant when the samples were irradiated sequentially with Ar^+^ + He^+^ + D^+^. Additionally, the two alloys presented opposite behaviors in relation to deuterium retention: while the (CrFeTiTa)_70_W_30_ sample presented 5.47% deuterium retention when implanted with He^+^ +D^+^ and 14.41% when implanted with Ar^+^ + He^+^ + D^+^, the VFeTiTaW alloy showed a decrease from 13.20% to 8.58% deuterium retention when it was implanted with Ar^+^ + He^+^ + D^+^. While the increase in deuterium retention in the (CrFeTiTa)_70_W_30_ sample can be attributed to the formation of defects that act as traps, the decrease observed in the VFeTiTaW sample remains unexplained and requires further investigation to explain the mechanism. The VFeTiTaW alloy thus presents lower deuterium retention when the effect of Ar^+^ is considered. It is, therefore, concluded that both alloys are promising for nuclear fusion applications, with VFeTiTaW presenting the most promising results. Moreover, studies on these alloys to establish mechanical property variations with irradiation should be considered for future work.

## Figures and Tables

**Table 1:** Irradiated samples.

Composition	Implanted Ions		
	Ar^+^	He^+^	D^+^
(CrFeTiTa)_70_W_30_	x		
(CrFeTiTa)_70_W_30_		x	x
(CrFeTiTa)_70_W_30_	x	x	x
VFeTiTaW	x		
VFeTiTaW		x	x
VFeTiTaW	x	x	x

**Table 2:** Average elemental composition (at.%) of phases (B) and (C) present in (CrFeTiTa)_70_W_30_ alloy annealed at 1500 °C and phases (E) and (F) present in VFeTiTaW alloy annealed at 1500 °C determined by point EDS analysis.

**(CrFeTiTa)_70_W_30_**	**Cr (at.%)**	**Fe (at.%)**	**Ti (at.%)**	**Ta (at.%)**	**W (at.%)**
Phase **B**	22.5 ± 0.5	49.0 ± 0.3	-	20.8 ± 0.1	7.7 ± 0.3
Phase **C**	-	-	2.6 ± 1.9	21.1 ± 0.1	76.3 ± 1.8
**VFeTiTaW**	**V (at.%)**	**Fe (at.%)**	**Ti (at.%)**	**Ta (at.%)**	**W (at.%)**
Phase **E**	27.3 ± 0.2	40.6 ± 0.3	-	25.4 ± 0.1	6.7 ± 0.4
Phase **F**	17.8 ± 0.4	-	-	17.2 ± 0.4	65.1 ± 0.4

**Table 3:** Deuterium retention on (CrFeTiTa)_70_W_30_ and VFeTiTaW alloys after He^+^ + D^+^ irradiation, and Ar^+^ + He^+^ + D^+^ irradiation. The values were determined using SIMNRA software.

	Implanted with He^+^ + D^+^	Implanted with Ar^+^ + He^+^ + D^+^
(CrFeTiTa)_70_W_30_	5.47%	14.41%
VFeTiTaW	13.20%	8.58%

## Data Availability

The original contributions presented in the study are included in the article; further inquiries can be directed to the corresponding author.
